# Validation with single-step SNPBLUP shows that evaluations can continue using a single mean of genotyped individuals, even with multiple breeds

**DOI:** 10.1186/s12711-023-00787-1

**Published:** 2023-03-22

**Authors:** Michael Aldridge, Jeremie Vandenplas, Pascal Duenk, John Henshall, Rachel Hawken, Mario Calus

**Affiliations:** 1 Animal Breeding and Genomics, Wageningen University and Research, P.O. Box 338, 6700 AH Wageningen, The Netherlands; 2 Cobb-Vantress Inc., Siloam Springs, AR 72761‑1030 USA

## Abstract

**Background:**

In genomic prediction, it is common to centre the genotypes of single nucleotide polymorphisms based on the allele frequencies in the current population, rather than those in the base generation. The mean breeding value of non-genotyped animals is conditional on the mean performance of genotyped relatives, but can be corrected by fitting the mean performance of genotyped individuals as a fixed regression. The associated covariate vector has been referred to as a ‘J-factor’, which if fitted as a fixed effect can improve the accuracy and dispersion bias of sire genomic estimated breeding values (GEBV). To date, this has only been performed on populations with a single breed. Here, we investigated whether there was any benefit in fitting a separate J-factor for each breed in a three-way crossbred population, and in using pedigree-based expected or genome-based estimated breed fractions to define the J-factors.

**Results:**

For body weight at 7 days, dispersion bias decreased when fitting multiple J-factors, but only with a low proportion of genotyped individuals with selective genotyping. On average, the mean regression coefficients of validation records on those of GEBV increased with one J-factor compared to none, and further increased with multiple J-factors. However, for body weight at 35 days this was not observed. The accuracy of GEBV remained unchanged regardless of the J-factor method used. Differences between the J-factor methods were limited with correlations approaching 1 for the estimated covariate vector, the estimated coefficients of the regression on the J-factors, and the GEBV.

**Conclusions:**

Based on our results and in the particular design analysed here, i.e. all the animals with phenotype are of the same type of crossbreds, fitting a single J-factor should be sufficient, to reduce dispersion bias. Fitting multiple J-factors may reduce dispersion bias further but this depends on the trait and genotyping rate. For the crossbred population analysed, fitting multiple J-factors has no adverse consequences and if this is done, it does not matter if the breed fractions used are based on the pedigree-expectation or the genomic estimates. Finally, when GEBV are estimated from crossbred data, any observed bias can potentially be reduced by including a straightforward regression on actual breed proportions.

**Supplementary Information:**

The online version contains supplementary material available at 10.1186/s12711-023-00787-1.

## Background

In genomic prediction, genotypes of single nucleotide polymorphisms (SNPs) are usually centred, preferably using allele frequencies in the base generation [1]. Several methods have been proposed to correct for the fact that often the allele frequencies in the current population are used, because animals from the assumed base population are typically not genotyped. Vitezica et al. [2] and Fernando et al. [3] suggested that the mean breeding value of non-genotyped animals is conditional on the mean performance of genotyped relatives. The method proposed by Hsu et al. [4] corrects for the centring of genotypes based on observed allele frequencies, by fitting the mean performance of genotyped individuals as a fixed regression, and is similar to the method of Vitezica et al. [2] except that they considered a random regression instead. The associated covariate vector has been colloquially referred to as the ‘J-factor’. The J-factor for genotyped animals is equal to − 1, and for ungenotyped animals ranges from − 2 to 0, where animals that are ungenotyped but closely related to genotyped animals will have a J-factor value closer to − 1. Recently, the method of Hsu et al. [4] has begun to be implemented in practice. It has been shown to increase the accuracy of breeding values and to reduce their dispersion bias, particularly in populations where genotyping is predominantly done in the more recent generations or with heavy selective genotyping based on phenotypic performance [4]. To date, the described applications are mostly limited to data involving a single breed or population, see for example Vandenplas et al. [5], Vandenplas et al. [6], and Bermann et al. [7].

Several studies have explored the impact of fitting breed-specific models on the realized accuracy of prediction. An increase in accuracy of prediction of 2% and 3% (milk yield and milk protein, respectively) has been observed in dairy cattle [8]. For some specific traits and breeds, there might be some benefit for the accuracy of prediction [9], or when selective genotyping is not accounted for [10]. However, generally the benefit of fitting breed-specific allele frequencies is limited or nil [8–11]. Crossbred animals represent a special case, as each breed has a different base population, and the contributions of each breed may vary among the crossbred animals. Therefore, especially in the presence of selective genotyping, it may be important to properly consider this variation in genetic background in the model.

We found a single published example of a separate J-factor being fitted per breed but no indication of whether there was any benefit [12]. Our first aim for this study was to determine if fitting a J-factor for each breed in a crossbred population would have any benefit on accuracy of prediction or its dispersion bias, with a particular focus on when selective genotyping is applied. Our second aim was to explore if using pedigree-based expected versus observed breed fractions to calculate the multiple J-factors would have any effect on the estimates of the accuracy and dispersion bias of sire GEBV.

## Methods

This study reuses data from a broiler three-way cross experiment that was previously analysed [13–16]. The main advantages of this dataset are that (1) all the phenotyped animals are genotyped, (2) the pedigree and the observed breed composition of all the crossbred animals were previously derived by Calus et al. [13], and (3) the three purebred lines have been shown to be distantly related [15, 16]. Furthermore, Duenk et al. [14] analysed the same dataset using models based on the breed-of-origin (BOA) of alleles, which enabled us to compare our results to those obtained with that model. The two traits of interest in their study were: body weight recorded at approximately 7 (BW7) and 35 days (BW35), which we also used here because of data availability and for comparison with the findings of Duenk et al. [14].

To determine if there is any benefit of estimating separate coefficients of regressions on J-factors per breed, we tested three methods of estimating J-factor coefficients: (1) by fitting a single J-factor for all breeds (ONE), which is most similar to the current practice; (2) by estimating three J-factors from the pedigree-based expected breed fractions (EXP); and (3) by estimating three J-factors based on the observed breed fractions derived from the earlier BOA analysis (OBS). For each of these methods, we investigated if there was any improvement in accuracy or dispersion bias of prediction by using different genotyping rates, either by selecting animals at random or based on their phenotype. The full details on how the J-factors were estimated and how the results were calculated are defined later.

### Estimation of the covariate vector ${\mathbf{J}}$

We followed the method proposed by Hsu et al. [4], where a covariate is fitted to model the mean of the unselected base population. The value of this covariate for all animals is stored in a vector ${\mathbf{J}}$, where entries for genotyped individuals are equal to − 1. For ungenotyped individuals, the covariate is estimated as ${\mathbf{J}}_{n} = - {\mathbf{A}}_{ng} {\mathbf{A}}_{gg}^{ - 1} {\mathbf{1}}$, where ${\mathbf{A}}_{ng}$ and ${\mathbf{A}}_{gg}$ are the pedigree-based relationship submatrices of non-genotyped $(n)$ with genotyped $(g)$ individuals and only genotyped individuals, respectively. Ungenotyped individuals will have covariates that range from − 2 to 0, and ungenotyped individuals with no genotyped relatives will have covariates equal to 0. An in-house software package has been developed to implement the method of Tribout et al. [17] to efficiently compute the ${\mathbf{J}}$ vector. For the ungenotyped animals that are not ancestors of genotyped animals, the parent averages of the J-factor covariates are used and processed from the oldest to the youngest.

For both EXP and OBS, the method described above was extended to support multiple J-factors, as ${\mathbf{J}}_{n} = - {\mathbf{A}}_{ng} {\mathbf{A}}_{gg}^{ - 1} {\mathbf{Q}}$, where ${\mathbf{Q}}$ contains the breed fractions with the dimensions $g \times b$, with $g$ being the number of genotyped animals and $b$ the number of breeds (i.e., $b = 3$ in this study). For purebred sires and dams in EXP, the corresponding entries in ${\mathbf{Q}}$ are 1 in the column for the relevant purebred breed (the three breeds are denotated as A, B, or C), and 0 for the other two columns. Based on the expected breed fractions in this study, the crossbreds had entries in ${\mathbf{Q}}$ of 0.5 in the column corresponding to the sire breed A, and 0.25 in both columns corresponding to the dam breeds B and C.

### Dataset

We used a dataset on a broiler three-way cross experiment that was provided by Cobb Vantress. The original dataset included 161 sires from breed A. In total, 156 sires were mated with both purebred breed A dams and F1 dams (BC). A principal component analysis of pureline and crossbred genotypes by Duenk et al. [15] demonstrated that the three purebred breeds (A, B, and C) were genetically separate. A single generation of purebred and crossbred offspring were hatched across five consecutive batches between June 2014 and November 2014. In each batch, the offspring were housed in three to five pens. In total, 20 pens were used across the five batches, with near equal sex ratios in each batch. In 16 of the pens, the offspring were more than 90% purebred (A) or more than 90% crossbred (A(BC)), and in the remaining four pens the proportions of purebred vs crossbred were lower ranging from 53 to 77%. The majority of the offspring of a sire were housed together in the same pen, but each pen was represented by multiple sires.

For the analysis, we considered phenotype information from the crossbred offspring only. At approximately 7 and 35 days of age, all the animals were weighed. A previous outlier analysis that was performed by Duenk et al. [14] removed animals that deviated more than 3.5 standard deviations from the mean per day of recording. After these data edits, 10,585 records for BW7 (originally 10,602), and 10,272 records for BW35 days (originally 10,290) remained (Table 1). In Duenk et al. [14], purebred performance was also evaluated, but the number of purebred offspring was significantly smaller (4687 and 4471 records for BW7 and BW35, respectively). In our study, we did not use the purebred offspring. However, in the study of Duenk et al. [14], the number of crossbred progeny were filtered to reflect the number of purebreds in order to ensure a fair comparison between purebred and crossbred information. This was done by selecting full-sib families based on family size. In an initial analysis, Duenk et al. [14] showed that depending on which full-sib families were randomly selected, the results of the validation changed significantly. The random sampling in Duenk et al. [14] was repeated 100 times, which resulted in approximately 5000 crossbred progeny per replicate, and in our study, we used these same 100 sets as validation.

**Table 1:** Summary statistics for body weight at 7 (BW7) and 35 days of age (BW35)

Trait	Number of crossbred progeny^a^	Number of sires	Number of dams	Min	Mean ± sd	Max
BW7	10,585	156	1028	100	179 (23)	260
BW35	10,272	156	1027	922	2090 (302)	3097

^a^All crossbred progeny were phenotyped and used for variance component estimation, the 100 validation sets are selected from these animals

Standard deviations (sd) are provided in parentheses

For each of the 100 replicates, five cross-validation folds were created. Sires were selected such that there were four groups of 32 animals and one group of 33 animals, whose crossbred offspring were removed, in turn, from the reference population. These cross-validation folds were used to limit the relationships between the animals in the reference population and those in the validation population. For further information on how full-sib families were selected to subset the crossbred data and on how sires were selected for the validation replicates and cross-validation folds, see Duenk et al. [14].

All the crossbred animals were genotyped with a custom 60k Illumina SNP chip [18]. After removal of SNPs with low call rates, SNPs with minor allele frequencies lower than 0.005, and SNPs with parent–offspring inconsistencies greater than 1% based on derived parentage, 50,960 SNPs remained for further analyses. For the different analyses, four genotyping rates were considered (100, 75, 50 and 25%). The animals with a genotype that were included in the analyses were selected either randomly or based on phenotypic performance to mimic random genotyping or selective genotyping, respectively. In the scenario with random genotyping (RND), the required proportion of animals was randomly selected from the reference population and their genotypes were removed. For scenarios that considered selective genotyping based on phenotypic performance (TOP), the required proportion of animals with a genotype was selected based on their own observation from the top performing animals for the trait of interest. The genotypes of unselected animals were discarded. Selection was implemented independently for each trait, resulting in different groups of animals with a genotype in the TOP scenarios for BW7 and BW35. Selection of animals for both RND and TOP was performed within each cross-validation fold.

### Computation of observed breed fractions

To compute multiple J-factors, it is necessary to use the contribution of each breed. We derived the breed fractions from a previous BOA analysis [13], based on the approach of Vandenplas et al. [19]. We decided on this method because the previously derived BOA were available and because this is probably one of the most accurate ways of determining the actual breed composition [20].

Across the three breeds, the sum of the breed fractions for each individual is expected to equal 1. The contributions of the sire breed to crossbreds were fixed at 0.5, because that is the exact contribution of a sire breed to a 3-way crossbred. The BOA analysis achieved a close 0.495 of the sire breed contribution. The contributions of the two dam breeds across all animals were first corrected for each dam breed, separately, such that the mean proportion across all the crossbreds is equal to the expected average value of 0.25 for both dam breeds. Then, for each crossbred animal, the contributions of each of the two dam breeds were scaled to ensure that their combined contribution was 0.5. For both dam breeds, the scaled contributions ranged from 0.09 to 0.41.

### Genomic prediction using J-factors

All variance components were estimated by restricted maximum likelihood (REML) using a univariate animal model in the ASReml program version 4.1 [21]. For both traits, BW7 and BW35, the model can be summarized in matrix notation as:

1 $${\mathbf{y}} = {\mathbf{Xb}} + {\mathbf{Zu}}_{{\mathbf{p}}} + {\mathbf{Wc}} + {\mathbf{e}},$$

where ${\mathbf{y}}$ is the vector of observations, ${\mathbf{X}}$, ${\mathbf{Z}}$, and ${\mathbf{W}}$ are incidence matrices that relate phenotypes to the vectors of fixed, additive genetic, and non-genetic permanent environmental effects, respectively, i.e. ${\mathbf{b}}$ is the vector of fixed effects (batch × pen × sex × age in days) with 52 levels for BW7 and 72 levels for BW35, ${\mathbf{u}}_{{\mathbf{p}}} \sim {\text{MVN}}\left( {{\mathbf{0}},{\mathbf{A}}\sigma_{a}^{2} } \right)$ is the vector of additive genetic effects, and ${\mathbf{c}}\sim {\text{MVN}}\left( {{\mathbf{0}},{\mathbf{I}}\sigma_{{\text{c}}}^{2} } \right)$ is the vector of non-genetic maternal permanent environmental effects, and ${\mathbf{e}}\sim {\text{MVN}}\left( {{\mathbf{0}},{\mathbf{I}}\sigma_{{\text{e}}}^{2} } \right)$) is a vector of residuals. The terms $\sigma_{{\text{a}}}^{2}$, $\sigma_{{\text{c}}}^{2}$, and $\sigma_{{\text{e}}}^{2}$, are the variances for additive genetic, non-genetic maternal permanent environment, and residual effects, respectively, with ${\mathbf{A}}$ and ${\mathbf{I}}$ being a pedigree-based relationship matrix (18,177 and 17,640 animals for BW7 and BW35, respectively) and an identity matrix, respectively.

Unlike in the previous estimation of variance components using this dataset [15], we considered only crossbred information and the pedigree-based relationship matrix. The estimates of heritability were similar to those previously used, i.e., 0.26 for BW7 and 0.25 for BW35. The variance components estimated with this pedigree-based model (Table 2) were used as the variance components of the random effects in all the single-step SNP best linear unbiased prediction (ssSNPBLUP) models. Note that the scenarios with a 100% genotyping rate are still considered as ssSNPBLUP, since some ancestors in the pedigree are not genotyped.

**Table 2:** Variance components used for genomic prediction: additive genetic $\left( {\sigma_{{{\text{u}}_{{\text{p}}} }}^{2} } \right)$, non-genetic maternal permanent environment $\left( {\sigma_{{\text{c}}}^{2} } \right)$, residual $\left( {\sigma_{{\text{e}}}^{2} } \right)$, and the estimated heritability $\left( {h^{2} } \right)$

Trait	$\sigma_{{u_{p} }}^{2}$	$\sigma_{c}^{2}$	$\sigma_{{\text{e}}}^{2}$	$h^{2}$
BW7	69.01 (6.66)	43.73 (10.18)	151.07 (25.41)	0.26 (0.04)
BW35	8,240.59 (7.33)	1,235.72 (3.59)	24,163.40 (33.73)	0.25 (0.03)

Standard errors in parentheses

For each of the J-factor approaches (ONE, EXP, and OBS), each of the selective genotyping (TOP, RND) methods, each genotyping rate (100, 75, 50 and 25%), and each cross-validation fold within validation replicates, GEBV were predicted using the hpblup solver in MiXBLUP [22]. For this purpose, a ssSNPBLUP model following the method of Liu et al. [23] with one covariate for the J-factor in ONE and three covariates in EXP and OBS was as follows:

2 $${\mathbf{y}} = {\mathbf{Xb}} + {\mathbf{ZJ{\mathbf{\upmu}}}}_{{\varvec{g}}} + {\mathbf{Zu}}_{{\mathbf{s}}} + {\mathbf{Wc}} + {\mathbf{e}},$$

where ${\mathbf{J}}$ is the previous estimated covariate matrix for EXP and OBS and a vector for NONE, and ${{\mathbf{\upmu}}}_{{\varvec{g}}}$ is a vector with the mean performance for each breed of genotyped animals for EXP and OBS, and a scalar for ONE, and the additive genetic effects of genotyped animals:

$${\mathbf{u}}_{{\mathbf{s}}} = {\mathbf{Z}}_{g} {\mathbf{g}} + {\mathbf{a}},$$

where ${\mathbf{Z}}_{g}$ is the matrix of centred SNP genotypes for genotyped animals, ${\mathbf{g}}$ is the vector of the additive genetic effects of the fitted SNPs, and ${\mathbf{a}}$ is the vector of residual polygenic effects. The observed allele frequencies across all genotyped animals were used to centre the SNP genotypes. Following Liu et al. [23], it follows that:

$$\begin{aligned} {\mathbf{u}}_{{\text{s}}} & = \left[ {\begin{array}{*{20}c} {{\mathbf{u}}_{s,n} } \\ {{\mathbf{u}}_{s,g} } \\ {\mathbf{g}} \\ \end{array} } \right] \\ & \sim {\text{MVN}}\left( {{\mathbf{0}},\left[ {\begin{array}{*{20}c} {{\mathbf{A}}_{nn} - {\mathbf{A}}_{ng} {\mathbf{A}}_{gg}^{ - 1} {\mathbf{A}}_{gn} + {\mathbf{A}}_{ng} {\mathbf{A}}_{gg}^{ - 1} {\mathbf{GA}}_{gg}^{ - 1} {\mathbf{A}}_{gn} } & {{\mathbf{A}}_{ng} {\mathbf{A}}_{gg}^{ - 1} {\mathbf{G}}} & {{\mathbf{A}}_{ng} {\mathbf{A}}_{gg}^{ - 1} {\mathbf{Z}}_{g} {\mathbf{B}}} \\ {{\mathbf{GA}}_{gg}^{ - 1} {\mathbf{A}}_{gn} } & {\mathbf{G}} & {{\mathbf{A}}_{ng} {\mathbf{A}}_{gg}^{ - 1} {\mathbf{Z}}_{g} {\mathbf{B}}} \\ {{\mathbf{BZ}}_{g}^{\prime } {\mathbf{A}}_{gg}^{ - 1} {\mathbf{A}}_{gn} } & {{\mathbf{BZ}}_{g}^{\prime } {\mathbf{A}}_{gg}^{ - 1} {\mathbf{A}}_{gn} } & {\mathbf{B}} \\ \end{array} } \right]{\varvec{\sigma}}_{u}^{2} } \right), \\ \end{aligned}$$

where $${\mathbf{A}} = \left[ {\begin{array}{*{20}c} {{\mathbf{A}}_{nn} } & {{\mathbf{A}}_{ng} } \\ {{\mathbf{A}}_{gn} } & {{\mathbf{A}}_{gg} } \\ \end{array} } \right]$$ is the pedigree-based relationship matrix between ungenotyped (“$n$”) and genotyped (“$g$”) animals, ${\mathbf{B}} = {\mathbf{I}}\frac{1 - w}{{2\sum p_{o} \left( {1 - p_{o} } \right)}}$, with $p_{o}$ being the observed allele frequency of the $o$-th SNP and $w$ being the proportion of variance (due to additive genetic effects) considered as residual polygenic effects, and ${\mathbf{G}} = {\mathbf{ZBZ^{\prime}}} + w{\mathbf{A}}_{gg}$ is the genomic relationship matrix among genotyped animals. In these analyses, the proportion of additive genetic variance $w$ considered to be due to residual polygenetic effects was assumed to equal 5%, and the estimated co-variance components used for the ssSNPBLUP evaluations were the same as the estimated co-variance components used for the pedigree-based BLUP evaluations. For further details on the ssSNPBLUP evaluation, see Vandenplas et al. [5, 6]. Finally, the GEBV analysed in this study were equal to ${\mathbf{J}}{\hat{\mathbf{\upmu}}} + {\hat{\mathbf{u}}}_{{\mathbf{s}}} .$

The same model without including ${\mathbf{J}}$ or ${{\varvec{\upmu}}}_{{\varvec{g}}}$ was used with a 100% genotyping rate to compare the effect of fitting no J-factor (NONE). However this is redundant because the J-factor in the scenario in which 100% of the phenotyped animals are genotyped is completely confounded with the general mean, and therefore only the results for a 100% genotyping rate with ONE are presented.

For each of the 100 replicates and five cross-validation folds, there were 16 scenarios. Each possible combination of genotyping rate, method of selective genotyping, and J-factor calculation was included in the genomic prediction (Table 3).

**Table 3:** Summary of the factors used in combination to build the scenarios

Scenario factors	Factor levels and abbreviations
Genotyping rate	100%, 75%, 50%, or 25%
Selective genotyping	No selective genotyping (RND) or genotyping based on phenotype (TOP)
J-factor calculation	No J-factor (NONE), one (ONE), observed (OBS), or expected (EXP) J-factor

### Accuracy and dispersion bias

Accuracies and dispersion bias of sire GEBV were computed using mean offspring performance records for both BW7 and BW35. The mean offspring performances records were computed by Duenk et al. [14] by averaging for each sire the offspring phenotypic records corrected for systemic environmental effects estimated with a sire model. More details on the computation of the mean offspring performance records are in Duenk et al. [14]. The accuracy of sire GEBV were estimated as a weighted correlation between the sire GEBV (estimated with the above genomic prediction) and the mean offspring performance. Dispersion bias was estimated as a weighted regression of the mean offspring performance on the same sire GEBV and multiplied by 2 so that the expectation is for an individual (1.00) rather than for a sire. The reliabilities of the mean offspring performance were used as the weights in the validation correlation and regression calculations. These reliabilities were estimated with the same method as in Duenk et al. [14] and following Cameron [24] as:

$$\frac{{\frac{1}{4}nh^{2} }}{{1 + \frac{1}{4}\left( {n - 1} \right)h^{2} }},$$

where $n$ is the number of offspring with records (between 2 and 430 for BW7 and between 2 and 404 for BW35) and $h^{2}$ is the estimated heritability (Table 2). The reliabilities ranged from 0.12 to 0.97 for BW7, and from 0.12 to 0.96 for BW35.

As the estimated slopes of the regressions on the J-factors differ for each cross-validation, the resulting GEBV may differ on scale. Therefore, the validation correlations and regression coefficients were estimated within cross-validation folds, which contrasts with the study of Duenk et al. [14] in which they were estimated across cross-validation folds within a replicate.

## Results

All of the results presented here are for the same 100 replicates and their cross-validation folds.

### Comparison of computed J factor covariates

With the J-factor method OBS, the mean J-factor covariates of phenotyped animals differed between the two dam breeds (Table 4) regardless of the selective genotyping method used. In contrast, the mean J-factor covariate of phenotyped animals for the EXP method were identical for the two dam breeds (Table 4). The differences between mean sire covariates across EXP and OBS were large, while those between the same dam covariates across J-factor methods were much smaller (Table 4).

**Table 4:** Mean J-factor covariates of the 100 replicates, estimated for the three breeds with animals phenotyped for body weight at 7 and 35 days (BW7 and BW35)

Genotyping rate	J-factor method^a^	Selective genotyping	BW7			BW35		
			Sire	Dam B	Dam C	Sire	Dam B	Dam C
75%	EXP	RND	− 0.411	− 0.194	− 0.194	− 0.406	− 0.192	− 0.192
	EXP	TOP	− 0.411	− 0.193	− 0.193	− 0.406	− 0.192	− 0.192
	OBS	RND	− 0.239	− 0.202	− 0.186	− 0.228	− 0.200	− 0.184
	OBS	TOP	− 0.239	− 0.201	− 0.186	− 0.228	− 0.199	− 0.184
50%	EXP	RND	− 0.411	− 0.188	− 0.188	− 0.406	− 0.185	− 0.185
	EXP	TOP	− 0.411	− 0.186	− 0.186	− 0.406	− 0.185	− 0.185
	OBS	RND	− 0.239	− 0.195	− 0.180	− 0.228	− 0.193	− 0.178
	OBS	TOP	− 0.239	− 0.194	− 0.179	− 0.228	− 0.192	− 0.178
25%	EXP	RND	− 0.411	− 0.178	− 0.178	− 0.406	− 0.175	− 0.175
	EXP	TOP	− 0.411	− 0.176	− 0.176	− 0.406	− 0.174	− 0.174
	OBS	RND	− 0.239	− 0.185	− 0.171	− 0.228	− 0.182	− 0.168
	OBS	TOP	− 0.239	− 0.183	− 0.169	− 0.228	− 0.181	− 0.168

^a^J-factor methods used include: three J-factors calculated using for each breed the expected (EXP), or observed (OBS) breed contribution calculated using BOA from previous analysis [13]

The distributions of the J-factor covariates (results not shown) were identical for the J-factor method ONE regardless of whether genotyping was random or based on phenotype. For EXP and OBS, the sum of the J-factor covariates had an identical distribution to that for ONE, which is expected since ${\mathbf{Q}}*{\mathbf{1}} = {\mathbf{1}}$. The distributions of the J-factor covariates were similar for both Dam breed B and Dam breed C across the J-factor methods and selective genotyping method. For the J-factor method OBS, the two dam breeds had covariates that were normally distributed but more stretched than for EXP.

### Estimated J-factor regression coefficients

The estimated J-factor regression coefficients were similar across scenarios and methods. For EXP, the estimated coefficients for the two dam breeds were identical, regardless of the genotyping rate and with or without selective genotyping. The estimates between the two dam breeds with OBS were not identical but quite similar. With a genotyping rate of 100% and for the three J-factor methods (ONE, EXP, and OBS), the J-factors are highly confounded with the general mean, which indicates that the results are not reliable and thus they are not presented. The sire is highly confounded with the breed which is picked up in the general mean, which indicates that the results of the estimated regression for the sire breed for OBS and EXP in Tables 5 and 6 are not reliable. At lower genotyping rates, there were differences between EXP and OBS, both with random genotyping (Table 5) and selective genotyping (Table 6), however the results were still similar. Finally, the absolute values of the regression coefficients for the dam breeds increased considerably with selective compared to random genotyping.

**Table 5:** Mean estimated J factor regression coefficients of the 100 replicates, for body weight at 7 and 35 days (BW7 and BW35), with random genotyping

Genotyping rate	J-factor method^a^	BW7			BW35		
		Sire	Dam B	Dam C	Sire	Dam B	Dam C
75%	ONE^b^	4.64			25.80		
	EXP	− 121.30	4.57	4.57	− 1403.35	25.80	25.80
	OBS	− 121.24	7.46	0.90	− 1402.87	58.70	0.90
50%	ONE^b^	4.22			27.29		
	EXP	− 121.13	4.20	4.20	− 1403.16	27.16	27.16
	OBS	− 121.08	7.48	0.11	− 1402.68	62.24	− 16.18
25%	ONE^b^	5.22			39.34		
	EXP	− 121.33	5.22	5.22	− 1405.45	39.46	39.46
	OBS	− 121.30	8.38	1.43	− 1405.78	85.33	− 14.74

^a^J-factor methods used include: one for all breeds (ONE), three J-factors calculated using for each breed the expected (EXP), or observed (OBS) breed contribution calculated using BOA from previous analysis [13]

^b^The estimate for ONE is for a single estimate for the full population and not the sire breed

**Table 6:** Mean estimated J factor regression coefficients of the 100 replicates, for body weight at 7 and 35 days (BW7 and BW35), with selective genotyping based on phenotypic performance

Genotyping rate	J-factor method^a^	BW7			BW35		
		Sire	Dam B	Dam C	Sire	Dam B	Dam C
75%	ONE^b^	− 194.37			− 1999.56		
	EXP	− 57.72	− 194.33	− 194.33	− 753.68	− 1999.07	− 1999.07
	OBS	− 57.67	− 191.97	− 197.38	− 753.23	− 1965.18	− 2040.79
50%	ONE^b^	− 137.75			− 1330.02		
	EXP	− 78.47	− 137.79	− 137.79	− 991.49	− 1330.17	− 1330.17
	OBS	− 78.45	− 136.32	− 139.57	− 991.03	− 1284.36	− 1385.35
25%	ONE^b^	− 89.32			− 785.36		
	EXP	− 95.54	− 89.27	− 89.27	− 1178.14	− 785.43	− 785.43
	OBS	− 95.50	− 88.11	− 90.84	− 1178.49	− 729.79	− 850.37

^a^J-factor methods used include: one for all breeds (ONE), three J-factors calculated using for each breed the expected (EXP), or observed (OBS) breed contribution calculated using BOA from previous analysis [13]

^b^The estimate for ONE is for a single estimate for the full population and not the sire breed

### Accuracy of sire GEBV

For both BW7 and BW35 and with either random or selective genotyping, there was no difference in accuracy of sire GEBV when using the J-factor methods ONE, EXP, or OBS (Table 7). When genotyping was random, the accuracy for BW7 was the same regardless of whether a J-factor was fitted or not. When genotyping was based on phenotypic performance for BW7, the accuracy tended to increase when a J-factor was fitted. The opposite was observed for BW35 with TOP selective genotyping, i.e. the accuracy decreased when J-factors were used. This difference seems to increase with more stringent selective genotyping. For BW35 with random genotyping, the accuracy remained the same if J-factors were used or not. For both traits and genotyping strategies, the accuracy decreased with decreasing genotyping rates.

**Table 7:** Mean validation correlations of 100 replicates for bodyweight at 7 days and 35 days (BW7 and BW35), for each method of selective genotyping (RND or TOP)

Genotyping rate	J-factor method^a^	BW7^b^		BW35^b^	
		RND	TOP	RND	TOP
100%^c^	Duenk et al. [14]	0.16 (0.058)		0.26 (0.060)	
	ONE^c^	0.17 (0.055)		0.26 (0.061)	
75%	NONE	0.14 (0.059)	0.13 (0.055)	0.23 (0.059)	0.28 (0.056)
	ONE	0.14 (0.061)	0.14 (0.061)	0.24 (0.061)	0.26 (0.061)
	EXP	0.14 (0.060)	0.14 (0.059)	0.24 (0.061)	0.26 (0.061)
	OBS	0.14 (0.071)	0.14 (0.059)	0.23 (0.055)	0.26 (0.066)
50%	NONE	0.13 (0.063)	0.10 (0.048)	0.21 (0.064)	0.25 (0.057)
	ONE	0.13 (0.057)	0.11 (0.056)	0.21 (0.064)	0.21 (0.065)
	EXP	0.12 (0.057)	0.11 (0.056)	0.21 (0.062)	0.21 (0.064)
	OBS	0.13 (0.059)	0.11 (0.055)	0.21 (0.064)	0.20 (0.064)
25%	NONE	0.11 (0.061)	0.10 (0.049)	0.18 (0.072)	0.23 (0.055)
	ONE	0.11 (0.062)	0.11 (0.062)	0.18 (0.066)	0.19 (0.063)
	EXP	0.11 (0.064)	0.11 (0.057)	0.18 (0.066)	0.19 (0.062)
	OBS	0.11 (0.062)	0.11 (0.056)	0.18 (0.065)	0.19 (0.063)

^a^J-factor methods used include none (NONE), one for all breeds (ONE), three J-factors calculated using for each breed the expected (EXP), or observed (OBS) breed contribution calculated using BOA from previous analysis [13]

^b^Standard deviations in parentheses. Standard deviation can be calculated as ${{{\text{SD}}} \mathord{\left/ {\vphantom {{{\text{SD}}} {\sqrt n }}} \right. \kern-0pt} {\sqrt n }}$, where SD is the standard deviation and *n* is the number of replicates (100)

^c^For 100% genotyping with ONE, it is equivalent to fitting NONE, EXP, and OBS

### Dispersion bias of sire GEBV

Dispersion bias of sire GEBV was evaluated by the regression coefficient of offspring averages on breeding values of sires, multiplied by 2, with an expected value of 1 for unbiased breeding values (Table 8). The dispression bias for BW7 was quite strong for all scenarios. For BW7 with random genotyping, there was some reduction in dispersion bias when fitting at least one J-factor, and as the genotyping rate decreased the dispersion bias increased (i.e. the estimated regression coefficient decreased from 0.40 when 100% of the population was genotyped to 0.32 when only 25% of the population was genotyped). Compared to random genotyping, when genotyping was based on phenotypic performance for BW7, dispersion bias decreased, but it should be noted that considerable differences were found when fitting J-factors and between J-factor methods. At a genotyping rate of 75%, the estimated regression coefficient with no J-factors was 0.31, but when J-factors were included it increased to 0.40. Similarly at a genotyping rate of 50% with no J-factors the estimated regression coefficient was 0.24, but when J-factors were included it increased to 0.36. At the lowest genotyping rate (25%), the benefit of fitting multiple J-factors appeared to increase, i.e. when no J-factor was fitted the estimated regression coefficient was 0.27, with the J-factor from method ONE it increased slightly at 0.33, and with the J-factors from both the EXP and OBS methods, it further increased to 0.37.

**Table 8:** Mean regression coefficients of validation records on GEBV from 100 replicates for bodyweight at 7 days and 35 days (BW7 and BW35), with both methods of selective genotyping (RND or TOP)

Genotyping rate	J-factor method^a^	BW7^b^		BW35^b^	
		RND	TOP	RND	TOP
100%^c^	Duenk et al. [14]	0.36 (0.133)		0.64 (0.158)	
	ONE^c^	0.39 (0.142)		0.76 (0.190)	
75%	NONE	0.35 (0.163)	0.31 (0.142)	0.72 (0.191)	0.84 (0.166)
	ONE	0.37 (0.161)	0.40 (0.192)	0.73 (0.200)	0.92 (0.220)
	EXP	0.37 (0.160)	0.40 (0.186)	0.76 (0.203)	0.92 (0.220)
	OBS	0.36 (0.193)	0.40 (0.187)	0.70 (0.171)	0.91 (0.237)
50%	NONE	0.34 (0.185)	0.24 (0.132)	0.70 (0.221)	0.76 (0.178)
	ONE	0.35 (0.170)	0.36 (0.209)	0.69 (0.229)	0.78 (0.246)
	EXP	0.35 (0.174)	0.36 (0.209)	0.69 (0.218)	0.79 (0.244)
	OBS	0.35 (0.176)	0.36 (0.205)	0.69 (0.222)	0.78 (0.243)
25%	NONE	0.32 (0.199)	0.27 (0.149)	0.65 (0.255)	0.78 (0.188)
	ONE	0.33 (0.206)	0.37 (0.304)	0.65 (0.240)	0.77 (0.263)
	EXP	0.33 (0.215)	0.37 (0.224)	0.65 (0.240)	0.78 (0.260)
	OBS	0.33 (0.206)	0.37 (0.222)	0.65 (0.238)	0.78 (0.264)

^a^J-factor methods used include none (NONE), one for all breeds (ONE), three J-factors calculated using for each breed the expected (EXP), or observed (OBS) breed contribution calculated using BOA from previous analysis [13]

^b^Standard deviations in parentheses. Standard deviation can be calculated as ${{{\text{SD}}} \mathord{\left/ {\vphantom {{{\text{SD}}} {\sqrt n }}} \right. \kern-0pt} {\sqrt n }}$, where SD is the standard deviation and $n$ is the number of replicates (100)

^c^For 100% genotyping with ONE, it is equivalent to fitting NONE, EXP, and OBS

Compared to BW7, the differences in dispersion bias for BW35 were not as clear. Generally, the dispersion bias was reduced, with estimates of the regression coefficient ranging from 0.64 to 0.80 when genotyping was random. Again there was less dispersion bias at higher genotyping rates with estimates of the regression coefficient decreasing from 0.80 at 100% genotyping to 0.65 at 25% genotyping. Interestingly, at the higher 100% and 75% genotyping rates, fitting multiple J-factors compared to ONE J-factor resulted in some benefit. However, there was no consistency between the results with EXP and OBS, and both were associated with larger variation in dispersion bias between replicates. For BW35, dispersion bias decreased only when J-factors were fitted and when 75% of the animals were genotyped based on phenotypic performance, but not for genotyping rates of 25 and 50%.

## Discussion

The aim of our study was to investigate if there is any benefit in estimating separate coefficients of regression on J-factors per breed. We compared validation correlations and regression coefficients of validation records on sire GEBV, and found that there is some benefit in fitting at least one J-factor, with a reduction in dispersion bias and in some situations a small increase in accuracy of sire GEBV. In some situations where multiple J-factors are fitted, dispersion bias is further reduced. If this experimental three way-cross were used for breeding purposes, fitting a single J-factor should be sufficient. However, the usefulness of multiple J-factors may differ with other breeding designs and population structures.

### Fitting no J-factor versus fitting one or multiple J-factors

Generally, fitting or not fitting a J-factor, yielded no difference in accuracy of sire GEBV but, in some cases, there was a considerable reduction in dispersion bias, which follows the expectations of Hsu et al. [4]. A considerable reduction in dispersion bias was clearly observed for BW7 at each genotyping rate with selective genotyping based on phenotypic performance. However, for BW35 with genotyping based on phenotypic performance and for both BW7 and BW35 with random genotyping, the reduction in dispersion bias was minimal. The only improvement in dispersion bias for BW35 was at a genotyping rate of 100% when OBS was used. It should be noted that when 100% of the animals were genotyped, the NONE, ONE and EXP methods are equivalent, because in this case there is no variation in the J-factors across animals for ONE and EXP. It should also be noted that, for the dam breeds and for both traits, the estimated coefficients of the regression on J-factors for EXP were similar to those for ONE, regardless of whether genotyping is random or selective. Therefore, the variation captured by the EXP J-factors for the dam breeds, is represented in the variation of the ONE J-factor, which is probably much larger than the variation in J-factors due to the sire breed. As a result, the regression on the sire breed effectively models an intercept in the model, which is captured by the other fixed effects when using ONE. This means that, probably, only the estimates for the dam breeds are meaningful. There is variation captured by the OBS J-factors for the dam breeds, which indicates that the J-factors effectively model the variation that the two different dam breeds contribute, unlike EXP, ONE, and NONE.

For BW35, there was an issue when the low genotyping rate based on phenotypic performance was applied, since the accuracy was lower when fitting a J-factor; this tendency was also observed for BW7 but with much smaller differences. When not fitting a J-factor, the unaccounted differences between genotyped and non-genotyped animals would be included in the breeding values, leading to an inflated accuracy. This would also explain why the differences are larger with the more stringent selective genotyping, as in this case, it is more important to account for the differences between genotyped and non-genotyped animals. This explanation is supported by the fact that, the sum of the estimated coefficients for the dam breeds drops considerably with decreasing rates of genotyping (Table 6). With different genotyping rates, the difference in mean breeding value between genotyped and non-genotyped animals will change. However, we may expect a similar difference in mean breeding values between scenarios with genotyped and non-genotyped animals with 25 and 75% genotyped individuals, if the distribution of breeding values is more or less symmetric. However, it is expected that with a higher genotyping rate (75%), the model is better able to properly estimate the breeding values and mitigate the dispersion bias arising from the difference in mean breeding values between genotyped and non-genotyped animals that is not properly accounted for without fitting a J-factor.

When comparing the scenarios with no J-factors, including NONE, the scenario ONE with a 100% genotyping rate should also be considered because at this rate, a single J-factor is completely confounded with the general mean. That is why the estimates of accuracy and dispersion bias for NONE and ONE at a genotyping rate of 100% are identical. Thus, at this high genotyping rate it does not matter if a J-factor is fitted or not. Compared to the previous estimates of accuracy and dispersion bias from the same dataset reported by Duenk et al. [14] where no J-factors were used, with NONE we observed slightly higher estimates of accuracy and slightly less dispersion bias for BW7, and no difference in accuracy but less dispersion bias for BW35. While we used an identical cross-validation structure and the same animals in each cross-validation fold, unlike the previous analysis, we used parameter estimates that only considered crossbred information and the pedigree-based relationship matrix. The heritabilities were similar to the previously reported estimates of 0.26 for BW7 and 0.25 for BW35. The variance components estimated with this pedigree-based model (Table 2) were used as the variance components of the random effects in all of the ssSNPBLUP models. The largest difference between scenarios could be caused by the methods used to estimate accuracy and dispersion bias. While we used the same formulas and sire mean progeny performance values, in our analysis we were forced to calculate accuracy and dispersion bias within a cross-validation fold due to scale differences when fitting multiple J-factors, which were observed as parallel banding when plotted, whereas Duenk et al. [14] did not have this problem and calculated accuracy and dispersion bias within replicate.

We hypothesized that one of the reasons why there was a larger reduction in dispersion bias for BW7 when using ONE or multiple (EXP and OBS) J-factors compared to NONE, and why a similar reduction in dispersion bias was not observed for BW35, is that the maternal effect is more important for BW7 than for BW35. The model used, fitted a non-genetic maternal permanent environmental effect and if a maternal genetic component is not fitted, the results could be biased [25]. It is possible that by fitting separate covariates for the two maternal breeds, some maternal genetic information is captured, thereby reducing dispersion bias. However, we rejected this hypothesis because the estimated coefficients of the regression on J-factors for OBS were very similar for the two dam breeds. This could be tested by fitting a maternal genetic effect, using the pedigree information.

While there were some differences in the covariates estimated between the three J-factor methods (ONE, EXP, and OBS), there was limited benefit in fitting one J-factor or a separate J-factor per breed. We estimated covariates of J-factors for multiple breeds using two methods of calculating breed fractions, and as expected, the resulting covariates were very similar (see Additional file 1). Thus, it is not surprising that the resulting GEBV had a correlation close to 1, and that the estimates of accuracy and dispersion bias were very similar (within a standard error of ± 0.01). The estimated regression coefficients for J-factors of the two dam breeds were the same for EXP, because the covariates were the same, while the estimates for OBS were generally both close to those for EXP. The similarity of these estimates suggests that the created (phenotypic) difference between the genotyped and non-genotyped animals is similar for both dam breeds. Since the EXP breed fractions for the two dam breeds were identical, it indicates that it is possible to reduce the number of J-factors in some scenarios.

In our study, the population used was from an experimental design where a single generation of crossbreds (A(BC)) were hatched across five batches. The sires (A) and dams (BC) were selected from grandparents (of the crossbreds) from populations with divergent selection. It is likely that the impact of fitting breed-specific J-factors is population-dependent. We do not know what the effect of fitting multiple J-factors would be on other breeding designs, such as a three-way rotational cross or with multiple generations. Another reason why fitting multiple J-factors might be population-dependent (at least for OBS), is that the impact of the use of breed-specific allele frequencies in other applications was also found to be population-dependent [8–11]. It should be noted that in these previous applications, they were used to model breeding values rather than fixed effects. In our study, no adverse consequence was observed when using observed breed fractions calculated from BOA.

### Limitations

It should be noted that our study is limited by the population structure, i.e. all the animals with phenotype are of the same type of crossbreds, and generally speaking, very few datasets have complete phenotyping and genotyping of both purebred and crossbred animals, and with the breed contributions known. However, we are confident that the three breeds used here are sufficiently divergent for the usefulness of fitting multiple J-factors to be observable. This is based on the fact that these breeds have been selected from real breeding lines that are divergent, such that the average F_ST_ value between the three breeds is 0.24, which is greater than between simulated populations that separated 50 generations ago (see Calus et al. [16]). It would be interesting to consider other breeding designs and population structures, especially with multiple different types of crossbreds included, to investigate if they would benefit from additional J-factors.

The effect of fitting multiple J-factors could be trait-specific. We considered only two traits, both being body weight traits with high genetic correlations and similar heritabilities. While BW7 and to a lesser extent BW35 do have a maternal component, it would be interesting to consider other traits with a larger maternal component, especially when the dam breeds have been historically selected for maternal traits. The study of Bermann et al. [7] showed that fitting the mean of genotyped animals can increase accuracy and reduce dispersion bias if the genomic and pedigree relationship matrices are not scaled, whereas if they are scaled, fitting the mean of genotyped animals increases dispersion bias but has no effect on accuracy. In the same study, fitting the mean performance as a fixed or random effect was also examined, and it was found that fitting it as a random effect reduced dispersion bias whereas fitting it as a fixed effect increased it. We did not consider these factors in our analysis, but based on the results of Bermann et al. [7], further improvements could potentially be made by fitting the J-factors as a random effect.

Finally, although our aim was to be able to compare directly our results with those of Duenk et al. [14], we had to calculate results within a cross-validation fold rather than a replicate because of scaling differences with multiple J-factors. While at a genotyping rate of 100% our estimates of accuracy were slightly higher and had less dispersion bias, the fact that we had only 30 observations available for each calculation rather than 150 may have limited our estimates of accuracy and dispersion bias at lower genotyping rates. We suggest that future studies exploring multiple J-factors consider more varied traits, with more breeds and/or family groups, consider different crossbreeding systems, explore different modelling strategies such as multi-trait models or fitting J-factors as a random effect, and if possible increase the number of observations per validation fold.

## Conclusions

We performed a cross-validation study in a 3-way crossbred population using ssSNPBLUP and fitting J-factors or not, to determine if there is any benefit in fitting a specific J-factor for each breed compared to fitting a single J-factor. We found that fitting a single J-factor was easier and that it would be sufficient if this experimental data was used for breeding purposes, with a reduction in dispersion bias and possibly some increase in accuracy. Fitting multiple J-factors may further reduce dispersion bias but this appears to depend on the trait and the genotyping rate. If a similar population structure is used for breeding purposes and multiple J-factors are used, using breed fractions that are based on the expectation or observation has no impact. In applications where breeding values estimated from crossbred data are inflated, i.e. there is too much variance in the breeding values, it may be beneficial to include a straightforward regression on actual breed proportions, as we observed for our scenario with a 100% genotyping rate. This analysis provides a suitable framework for testing the usefulness of multiple J-factors and demonstrates the robustness of the single-step method when fitting multiple J-factors.

## Supplementary Information

**Additional file 1.** Mathematical proof that the covariates estimated with ONE J-factor are equal to the sum of covariates estimates from multiple J-factors regardless of whether the breed fractions used are from expectation (EXP) or observation (OBS).

## Data Availability

The data used in the present study were provided by Cobb-Vantress, Inc and are not publicly accessible. Raw phenotypes and genotypes are only available through Cobb-Vantress.
