# Supplementary material for: Validation with single-step SNPBLUP shows that evaluations can continue using a single mean of genotyped individuals, even with multiple breeds
Source: Genet Sel Evol. 2023 Mar 22;55:19. doi: 10.1186/s12711-023-00787-1 (PMC10031914; doi:10.1186/s12711-023-00787-1)
Supplement: Supplementary file 1 — Additional file 1. Mathematical proof that the covariates estimated with ONE J-factor are equal to the sum of covariates estimates from multiple J-factors regardless of whether the breed fractions used are from expectation (EXP) or observation (OBS). [file 12711_2023_787_MOESM1_ESM.docx]

To compute multiple J-factors based on observed (OBS) breed fractions we use $\mathbf{J}_{n,OBS}=-\mathbf{A}_{ng}\mathbf{A}_{gg}^{-1}\mathbf{Q}_{\mathrm{OBS}}$, where $\mathbf{Q}_{\mathrm{OBS}}$ contains the observed breed fractions with the dimensions *g* x *b*, with *g* being the number of genotyped animals and *b* the number of breeds. For the OBS scenario, genotyped crossbred *j* has observed breed fraction ${bf}_{j}$ for breed B, and observed breed fraction $0.5-{bf}_{j}$ for breed C. So, for *g* ungenotyped animals, we get:

$$\mathbf{Q}_{\mathrm{OBS}}=\left[ \begin{matrix} 0.5 & {bf}_{1} & 0.5-{bf}_{1} \\ 0.5 & {bf}_{2} & 0.5-{bf}_{2} \\ \vdots& \vdots& \vdots\\ 0.5 & {bf}_{g} & 0.5-{bf}_{g} \end{matrix} \right]$$

For convenience, we write:

$$\mathbf{X}_{ng}=-\mathbf{A}_{ng}\mathbf{A}_{gg}^{-1}$$

Thus:

$$\mathbf{J}_{n,OBS}=\mathbf{X}_{ng}\mathbf{Q}_{\mathrm{OBS}}$$

It follows that the J-factor covariates for ungenotyped animal *i*:

$$\mathbf{J}_{i,OBS}=\mathbf{x}_{ig}\mathbf{Q}_{\mathrm{OBS}}=\left[ \begin{matrix} 0.5\sum_{j=1}^{g} x_{ij} & \sum_{j=1}^{g} x_{ij}{bf}_{j} & 0.5\sum_{j=1}^{g} x_{ij}-\sum_{j=1}^{g} x_{ij}{bf}_{j} \end{matrix} \right]$$

Where $\mathbf{x}_{ig}$ is a 1 x *g* vector.

And the sum of the J-factor covariates for ungenotyped animal *i* is:

$$\begin{matrix} 0.5\sum_{j=1}^{g} x_{ij}+\sum_{j=1}^{g} x_{ij}{bf}_{j}+0.5\sum_{j=1}^{g} x_{ij}-\sum_{j=1}^{g} x_{ij}{bf}_{j}=\sum_{j=1}^{g} x_{ij} \end{matrix}$$

Which is not affected by the observed breed fractions, and equal to the J-factor covariate when not considering the different breeds:

$$\mathbf{J}_{n,ONE}=-\mathbf{A}_{ng}\mathbf{A}_{gg}^{-1}\boldsymbol{1}$$

And:

$$\mathbf{J}_{i,ONE}=\mathbf{x}_{ig}\mathbf{1}\boldsymbol{=}\sum_{j=1}^{g} x_{ij}$$

For EXP, we get:

$$\mathbf{Q}_{\mathrm{EXP}}=\left[ \begin{matrix} 0.5 & 0.25 & 0.25 \\ 0.5 & 0.25 & 0.25 \\ \vdots& \vdots& \vdots\\ 0.5 & 0.25 & 0.25 \end{matrix} \right]$$

$$\mathbf{J}_{n,EXP}=\mathbf{X}_{ng}\mathbf{Q}_{\mathrm{EXP}}$$

It follows that the J-factor covariates for ungenotyped animal *i*:

$$\mathbf{J}_{i,EXP}=\mathbf{x}_{ig}\mathbf{Q}_{\mathrm{EXP}}=\left[ \begin{matrix} 0.5\sum_{j=1}^{g} x_{ij} & 0.25\sum_{j=1}^{g} x_{ij} & 0.25\sum_{j=1}^{g} x_{ij} \end{matrix} \right]$$

And the sum of the J-factor covariates for ungenotyped animal *i* is:

$$\begin{matrix} 0.5\sum_{j=1}^{g} x_{ij}+0.25\sum_{j=1}^{g} x_{ij}+0.25\sum_{j=1}^{g} x_{ij}=\sum_{j=1}^{g} x_{ij} \end{matrix}$$
